# What Differentiates Rural and Urban Patients with Type 1 Diabetes—A Pilot Study

**DOI:** 10.3390/nu16010022

**Published:** 2023-12-20

**Authors:** Beata I. Sińska, Alicja Kucharska, Ewa Rzońca, Leszek Wronka, Grażyna Bączek, Robert Gałązkowski, Dominik Olejniczak, Patryk Rzońca

**Affiliations:** 1 Department of Human Nutrition, Faculty of Health Sciences, Medical University of Warsaw, 01-445 Warsaw, Poland; alicja.kucharska@wum.edu.pl (A.K.); leszek.wronka@wum.edu.pl (L.W.); 2 Department of Obstetrics and Gynecology Didactics, Faculty of Health Sciences, Medical University of Warsaw, 00-575 Warsaw, Poland; erzonca@wum.edu.pl (E.R.); grazyna.baczek@wum.edu.pl (G.B.); 3 Department of Emergency Medical Services, Faculty of Health Sciences, Medical University of Warsaw, 00-575 Warsaw, Poland; robert.galazkowski@wum.edu.pl; 4 Department of Public Health, Faculty of Health Sciences, Medical University of Warsaw, 02-007 Warsaw, Poland; dominik.olejniczak@wum.edu.pl; 5 Department of Human Anatomy, Faculty of Health Sciences, Medical University of Warsaw, 02-004 Warsaw, Poland; patryk.rzonca@wum.edu.pl

**Keywords:** diabetes, rural, urban, adults

## Abstract

The effective management of diabetes is a complex issue and may be determined according to numerous patient-dependent and patient-independent factors. This study aimed to analyze the relationship between the place of residence and selected sociodemographic, psychological and diabetes-related parameters in people with type 1 diabetes (T1D). This study was conducted on 419 adults with T1D using nonprobability sampling. The following questionnaires were used: the Diabetes Dietary Guidelines Adherence Index, the Acceptance of Illness Scale, the Sense of Responsibility for Health Scale, the Diabetes Eating Problem Survey-Revised scale, the Eating Attitudes Test and questions on sociodemographic and diabetes-related parameters. People living in rural areas were characterized by a significantly lower age and level of education, a higher incidence of being overweight, a higher glycated hemoglobin concentration, a lower number of glucose measurements during the day and a higher level of acceptance of the disease compared to urban residents. The degree of adherence to dietary recommendations and the sense of responsibility for one’s own health were significantly higher among urban residents. It is necessary to assess barriers to a proper diet and to increase the effectiveness in managing the disease in rural communities. Targeted actions promoting the health of type 1 diabetics need to be developed with particular emphasis on patients from rural areas.

## 1. Introduction

Diabetes is a dominant non-communicable disease posing a huge challenge to modern healthcare systems due to the constantly growing number of cases [1,2]. Type 1 diabetes (T1D), being a multifactorial and autoimmune disease, is characterized by the destruction of pancreatic β cells by T cells, which results in a deficiency of the synthesis and secretion of insulin [3,4]. A person diagnosed with diabetes needs to perform regular blood glucose control and monitoring, followed by adequate treatment, which means administering insulin using pens or pumps in the case of type 1 diabetes. In addition, lifestyle modifications, including proper diet, regular physical activity, weight reduction and smoking cessation, are important in T1D therapy. Acceptance of the disease by the patient and a high sense of responsibility for one’s own health are associated with better adherence to therapeutic recommendations and, consequently, have a positive effect on diabetes control, health and well-being [5,6]. Psychological aspects are crucial in the approach to the disease, therapy and self-management of diabetes, which does not change the fact that constant, regular care provided by a therapeutic team and permanent education and re-education of the patient are necessary for optimal results. In a situation where a patient is responsible for a large part of the duties related to therapy, constant contact with a physician/nurse/dietitian/physiotherapist provides strong support for the patient and plays an invaluable role in maintaining motivation [7]. The recommendations of scientific societies indicate that the education of a patient with diabetes does not end when they leave the physician’s office. The patient should be able to have constant and regular contact with the therapeutic team regardless of their place of residence [8,9]. The American Diabetes Association stated that diabetes care is frequently unsatisfactory. They also emphasized the paucity of cooperative, multidisciplinary teams that are prepared to teach adequate self-management to diabetes patients [9].

Diabetes is an essential health issue in the modern world, and its consequences affect millions of people. Therefore, factors related to its occurrence and treatment are the subject of numerous scientific studies [1,5,10,11,12,13]. The relationship between the health status of patients with diabetes and their place of residence is one of the studied areas [14,15]. It should be emphasized here that urbanization exerts a significant impact on lifestyle. It is also one of the driving forces of population health changes. On the one hand, this process contributes to better access to the resources of the healthcare system and education or social services. Conversely, due to negative lifestyle changes (increased intake of saturated fats and carbohydrates, sedentary lifestyles), the incidence of non-communicable diseases, especially obesity and diabetes, was observed to rise in relation to urbanization [14,15,16]. Furthermore, it was stated that the quality of care among rural community inhabitants is lower in comparison with the quality of care in urban communities, including access to specialized endocrine and diabetes care [17,18,19].

Undoubtedly, there are some differences between type 1 diabetics inhabiting rural and urban areas. However, the Polish literature lacks publications regarding the in-depth analysis of this issue. This study was conducted to better understand the reality of T1D therapy and self-management in terms of the place of residence with particular attention paid to the comparison of rural and urban areas.

This study aimed to analyze the relationship between the place of residence and selected sociodemographic, psychological and diabetes-related parameters in people with type 1 diabetes.

## 2. Materials and Methods

### 2.1. Participants and Study Design

**Figure 1:**
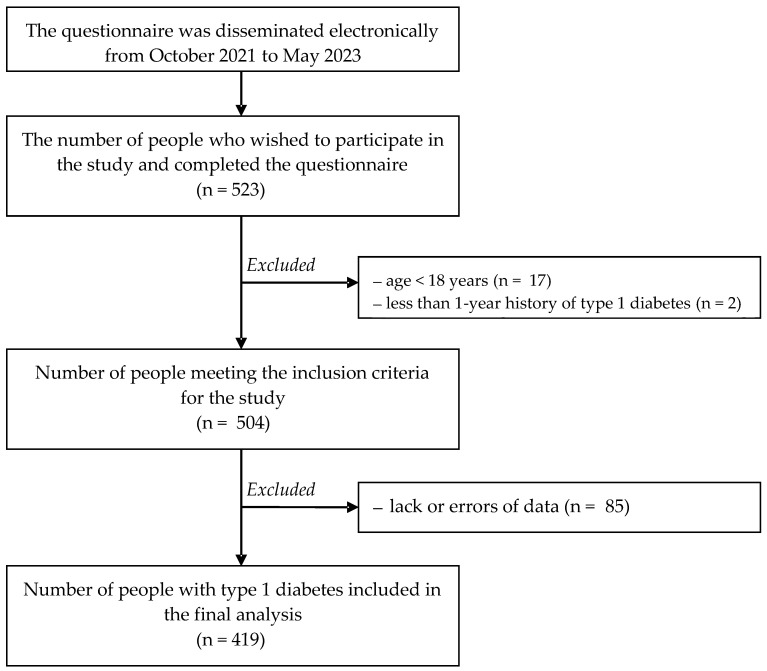
Flow diagram of the pilot study.

The timeframe of this study was from October 2021 to May 2023. The present authors used the diagnostic survey method with the online survey technique. A questionnaire was used as a research tool. The questionnaire was disseminated via the Google Forms web survey platform. Social media (Facebook) served as the channel for sharing the link to the questionnaire with patients with type 1 diabetes mellitus (groups: mojacukrzyca.org, CUKRZYCA 24 h INFO (DIABETES 24 h info), Cukrzyca typu 1—życie bez barier i na kolorowo (Diabetes mellitus type 1—life without barriers and in color)). We obtained the consent of site/group administrators. The inclusion criteria were as follows: age ≥ 18 years, a minimum of a 1-year history of type 1 diabetes and residence in Poland. Furthermore, the participants had to give their informed consent to be enrolled in the study. Eventually, the analysis included 419 individuals. Figure 1 presents a detailed scheme of the selection process.

Prior to the study, the participants were informed that it would be anonymous and the data would be confidential. No personal data were collected.

The research tool consisted of several parts. In the present authors’ part, we collected data concerning the sociodemographic aspects of the group of respondents (sex, education and place of residence) and diabetes-related information (i.e., body weight, height, current HbA1c value, method of insulin administration, duration of the disease, number of hypo- and hyperglycemic episodes, daily number of administered insulin units and insulin units per kg of body weight). All data were declared by the respondents. The second part of the questionnaire included the following:

− The Diabetes Dietary Guidelines Adherence Index (DDGA Index), which includes up-to-date recommendations on healthy eating and behavioral therapy guidelines issued by the Polish Diabetes Association. The index is used to assess the frequency of the consumption of 29 groups of products and meal regularity. The value of the DDGA index is expressed as the sum of the points obtained (0–30 points, where 0 points is non-compliance with the recommendations, and 1 point is compliance with the recommendations regarding the frequency of the consumption of a specific product group). Higher values of the DDGA Index are associated with a higher degree of adherence to dietary recommendations [20].
− The Acceptance of Illness Scale (AIS) questionnaire, based on Jurczyński’s adaptation, is composed of 8 statements that refer to the consequences of poor health status regarding the acceptance of limitations associated with the disease, no self-sufficiency, a sense of being dependent on others and a reduced sense of self-esteem. Each response is given a point value (strongly agree—1, strongly disagree—5). Scoring the lowest number of points (1) means a low level of adaptation to the disease, whereas strong disagreement (5 points) translates to disease acceptance. The total of 8–40 is a general outcome referring to the level of disease acceptance. With increasing acceptance, the degree of adaptation increases, and the sense of psychological discomfort decreases [21].
− The Diabetes Eating Problem Survey-Revised scale (DEPS-R) is a tool used to screen for eating disorders. The DEPS-R is diabetes-specific and is composed of 16 items. Respondents may select one of 6 responses on a 6-point Likert scale for each item. The total DEPS-R score ranges from 0 to 80 points. Higher overall DEPS-R scores indicate a higher likelihood of developing an eating disorder. According to the original version of the DEPS-R, a total score of 20 or above is assumed to be a threshold indicating a greater degree of disturbances [22].
− The short version of the Eating Attitude Test EAT-26 is a standardized nutritional attitude test used as a screening tool to assess the risk of eating disorders. Individuals who score 20 or more points in the test are more likely to develop an eating disorder [23].
− The Sense of Responsibility for Health Scale (SRHS), developed by Adamus [24], is composed of 14 items rated on a 5-point scale (1—hardly ever, 2—rarely, 3—sometimes, 4—often, 5—nearly always/very often). Only the total degree of responsibility for one’s health was subjected to evaluation in the present study. The Cronbach’s alpha for the scale was found to equal 0.724.

The application for the approval of the non-invasive study was submitted to and acknowledged by the Bioethics Committee of the Medical University of Warsaw.

### 2.2. Statistical Analysis

The data collected with the questionnaire were subjected to a statistical analysis using the STATISTICA software, version 13.2 (Tibco Software Inc., Palo Alto, CA, USA). Numbers (n) and percentages (%) were used to present categorical variables, and the mean (M) and standard deviation (SD) were used for continuous variables. The Kolmogorov–Smirnov and Lilliefors tests were used to verify the normal distributions of variables. Categorical and continuous variables were compared between urban and rural patients by using the chi-squared test and the Mann–Whitney U test, respectively. The threshold of statistical significance was assumed with a two-sided alpha level of 0.05.

## 3. Results

The statistical analysis showed that people with type 1 diabetes living in rural areas were significantly younger (M = 30.7 years) and more often had a secondary (54.1%) or primary/vocational (10.2%) education compared to urban residents (*p* < 0.05). No significant differences occurred between the sex and place of residence of the respondents (*p* > 0.05). Detailed data are presented in Table 1.

Based on the analysis of the Body Mass Index (BMI), it was found that healthy weight (55.8%) and obesity (1.6%) were more common in urban residents (*p* < 0.05) (Table 2).

The analysis revealed a significant difference between the place of residence of people with T1D and the concentration of glycated hemoglobin, as well as between the place of residence and the number of glucose measurements during the day (*p* < 0.05). Higher HbA1c scores (M = 7.8%) and taking glucose measurements more rarely than five times a day (33.7%) were more commonly found in respondents from rural areas. Detailed data on the selected diabetes-related parameters of people with T1D, taking account of the place of residence, are included in Table 3.

No significant differences were demonstrated between the place of residence of people with T1D and the use of psychological and psychiatric assistance (*p* < 0.05) (Table 4).

A higher degree of disease acceptance was more common among respondents from rural areas (M = 29.1 points). Conversely, a higher level of adherence to dietary recommendations specified in the DDGA Index (M = 18.0 points) and a higher sense of responsibility for one’s own health (M = 56.3 points) were more frequently found in respondents with T1D from urban areas (*p* < 0.05). Detailed data are included in Table 5.

## 4. Discussion

Numerous studies have demonstrated that the place of residence is an important factor differentiating health status and have indicated that populations from rural areas are characterized by poorer health indices [25,26,27,28]. This situation also applies to people with diabetes; poorer health outcomes are more often observed in patients living in rural areas [29,30,31]. Interesting research on health inequalities in eastern Poland was conducted by Pantyley. She reported that people with diabetes inhabiting rural areas and small towns have poorer health compared to people from urban areas. These results are justified by such factors as less attention to preventive measures, less and more difficult availability of specialist physicians (health centers are distant from the place of residence) and fewer financial resources of rural residents [32]. According to Bolin et al., diabetes constitutes the third most important health issue in rural communities in the USA [33]. In rural communities, diabetes is 8.6% more prevalent, and the morbidity and mortality associated with it are higher compared to urban areas [34,35]. The factors responsible for such a situation include a higher percentage of uninsured people, less access to healthcare, lower financial and educational status, lack of transport and complex factors related to an increased degree of overweight and obesity compared to the urban population [34]. Importantly, 62% of the rural population is devoid of access to programs promoting self-management techniques that are effective in improving diabetes management [36].

In the present study, an attempt was made to indicate those differences between the characteristics of people with T1D inhabiting urban and rural areas that could contribute to eliminating health inequalities. This study revealed that diabetics inhabiting rural areas were younger, and their level of education was lower compared to urban residents, which was also confirmed by Gill et al. [37].

Our research also provides evidence that people with type 1 diabetes inhabiting rural areas are significantly more likely to have an abnormal body weight (overweight and underweight) compared to people from towns/cities. Based on data available from the Central Statistical Office (GUS), no differences occurred in body weight among men from rural and urban populations in terms of deficiency (0.9% vs. 1.4%) and excessive body weight (61.4% vs. 62.7%). In the case of women, the percentage of individuals with body weight deficiency in urban and rural areas was similar: 4.3% and 4.1%, and overweight and obesity were found to occur more frequently in women from rural areas compared to those from towns/cities (47.4% vs. 44.6%) [38]. A normal body weight is linked to better metabolic control of diabetes. Research conducted by Jung et al. showed that the risk of death due to any cause is much higher in underweight patients with T1D than in those with normal body weights. Overweight and obese patients showed a heterogeneous risk in studies [39]. However, Edqvist et al. confirmed that the risk of mortality and the development of cardiovascular issues, especially among men, increases along with increasing mean BMI values in individuals with type 1 diabetes [40]. Some authors emphasized that normal body weights in patients with type 1 diabetes might be associated with a decreased risk of death, particularly in men [41]. An increased body weight in people with T1D may lead to so-called double diabetes, i.e., the simultaneous occurrence of the manifestations of type 1 and type 2 diabetes. According to research, rural residents are more at risk of developing diabetes because such areas are characterized by a higher prevalence of conditions such as obesity and metabolic syndrome [42,43].

Effective management of type 1 diabetes requires regular daily monitoring of blood glucose levels and adjusting the number of insulin doses. The present study showed no significant differences regarding the method of administering insulin (pen vs. pump), the frequency of hypo- and hyperglycemic episodes and insulin units per kilogram of body weight per day between urban and rural residents. However, it was demonstrated that people with T1D living in the countryside performed significantly fewer glucose measurements compared to urban residents. Glycemic fluctuations result in a high concentration of glycated hemoglobin, which contributes to a higher risk of diabetes complications experienced by a patient [18,44]. In this study, the parameter was significantly poorer in patients living in the countryside compared to people from towns/cities. Gill et al. reported similar observations. It was shown that the mean HbA1c concentration was markedly higher in rural patients in comparison with the urban group. The rural population was characterized by high significance despite being controlled in terms of potentially confounding differences between rural and urban residents [37]. The reasons for poorer diabetes control shown in rural residents analyzed in the present study may be linked to limited access to specialized medical care. Telemedicine might be helpful in improving this situation. Currently available data describing the impact of telemedicine on the treatment outcomes of patients with T1D are limited. However, a meta-analysis of 38 studies revealed a slightly positive effect of telemedicine on glycated hemoglobin concentrations [45]. Diabetes self-control also needs to be viewed within the framework of everyday roles and social contexts. The requirements of rural lifestyles are associated with irregular, not always predictable hours of professional and family responsibilities, which may contribute to non-compliance with certain recommendations, e.g., a lower frequency of glucose measurements. Although individuals with T1D may understand the importance of frequent/regular control of blood glucose levels and how to react in cases of hyperglycemia, they do not do so due to a number of responsibilities that have to be performed at the same time. An intense lifestyle may also influence whether a person is looking for additional information and skills to manage diabetes effectively [46,47].

Our own research shows that adherence to dietary recommendations was poorer among patients from rural communities, despite better acceptance of the disease. The results are in line with those obtained by Stumetz et al. They reported that poor adherence to attending appointments and medical recommendations, including dietary ones, appeared to be much poorer in respondents from rural areas [48]. Non-adherence to dietary recommendations by patients from rural areas may be due to the failure to take account of the specificity of cultural and nutritional norms in the proposed nutrition plans [47]. Patients want healthcare professionals to pay attention to their preferences when suggesting disease management strategies (including dietary recommendations). Such an approach is consistent with the recommendations of the Polish Society of Dietetics (2023), which indicate the need to take account of the individual nutritional and cultural preferences of the patient, as well as their age, sex, physical activity patterns and financial status. Recommendations should indicate the wide possibilities of individual choice and the composition of one’s diet. Importantly, nutrition education should be based on providing information in a practical way, enabling the application of the acquired knowledge directly in everyday life [8]. Providing dietary information on diabetes to the person mainly responsible for preparing meals and not only to the patient may be an important aspect supporting the implementation of dietary recommendations. Considering this aspect seems crucial, as roles were found to expand in rural communities, where two or more people in one household may work outside the home [49]. This study demonstrates that acceptance of the disease was more marked in rural inhabitants compared to urban ones. Acceptance of the disease is one of the most difficult stages of the disease process. It is believed that, with an increasing degree of acceptance, adaptation and life satisfaction levels increase, and the sense of mental discomfort decreases [21]. A similar observation was made by Kowalewska et al. in a study of patients with psoriasis, showing that the place of residence had an impact on life satisfaction (higher in rural residents), a sense of stigmatization (more marked in rural residents) and quality of life (greater deterioration in urban residents) [50]. Interestingly, as demonstrated in our study, higher acceptance of the disease in rural residents was not accompanied by a higher sense of responsibility for health or a higher degree of adherence to dietary recommendations. In addition, research results revealed that patients with diabetes are at a higher risk of developing mental health problems compared to people without the disease [51], and religious issues combined with self-acceptance could reduce anxiety disorders and depression in people with diabetes [52,53].

The differences observed in the present study indicate areas including barriers that had a negative influence on the self-control of the disease. Extending research in such areas may help with selecting appropriate and effective interventions and reduce health inequalities between rural and urban residents. Gap analysis and evaluations of available healthcare resources may contribute to developing a long-term vision for rural populations. It is worth considering the provision of support and offering practical solutions to people with T1D inhabiting rural areas, including a greater offer of online services and teleconsultation. Moreover, an opportunity to develop strong psychosocial support should be offered to all patients. Future research should focus on the needs of rural T1D patients to reduce disparities between urban and rural communities.

Children and adolescents with diabetes are included in the system of regional diabetes specialist care. Pediatric diabetes clinics are most often located in large urban centers. They are mainly found near provincial and university hospitals and are largely “connected” with diabetes wards, pediatric endocrinology or pediatric departments in these facilities. Conversely, care for adults with type 1 diabetes is provided by specialist diabetes clinics, primary care centers and departments of internal medicine, diabetes, endocrinology, surgery and others. In various regions of Poland, this structure is similar, but it may be focused on different aspects. Regrettably, it is also fragmented, i.e., often without a leading regional center that is well-equipped and can provide holistic care for adults with diabetes, including late complications. Access to specialized diabetes care, both on an outpatient and inpatient basis, is considerably more difficult for adults, which often contributes to the worsening of the metabolic control of diabetes. Restrictions in access to diabetes care quickly lead to the deterioration of health, especially in patients requiring an intensification of insulin therapy, developing complications and with diabetic foot syndrome. In some regions, there is a lack of well-equipped specialist clinics that provide diabetics with multi-level holistic care, including professional diabetes education, psychological support, advanced assessment and treatment of complications and the use of modern technologies [54].

The undoubted strength of this study is related to the fact that the issue was tackled in a group of individuals with type 1 diabetes, in a situation where most previous research has focused on type 2 diabetes patients. When looking for opportunities to improve disease control, factors that may contribute to it should be identified. The observations are also of practical significance in the context of developing and implementing educational interventions in this group of patients. This study is not devoid of limitations, with the main one being the small size of the study group and the convenient selection (the patients volunteered to participate in the survey themselves). The sample may not be deemed representative of the general population. Therefore, the results obtained may not be broadly generalized. We treat the conducted study as a pilot study that requires continuation in a larger group of patients. Additionally, we did not take account of cases in which people had just moved to the current rural area from an urban area or vice versa. Data on glycated hemoglobin concentrations and body weight and height were declared by the respondents. Anonymous surveys assume truthfulness and goodwill of the respondents, but obtaining such information via direct methods would be more valuable.

## 5. Conclusions

This pilot study showed differences between individuals with diabetes inhabiting urban and rural areas. It is necessary to assess barriers to a proper diet and to increase the effectiveness in managing the disease in rural communities. Further research should be conducted in order to develop targeted actions promoting the health of those suffering from type 1 diabetes, with particular emphasis on patients from rural areas.

## Figures and Tables

**Table 1:** Analysis of the relationship between the place of residence of type 1 diabetics and selected sociodemographic variables.

Variables	Total (n = 419)	Urban Area (n = 321)	Rural Area (n = 98)	*p*-Value
Age—M (SD) [years]	33.1 (10.7)	33.9 (11.3)	30.7 (7.9)	0.043
Sex—n (%)				
Woman	232 (55.4)	177 (55.1)	55 (56.1)	0.864
Man	187 (44.6)	144 (44.9)	43 (43.9)	
Education—n (%)				
Primary/vocational	37 (8.8)	27 (6.4)	10 (10.2)	0.006
Secondary	174 (41.5)	121 (37.7)	53 (54.1)	
Tertiary	208 (49.6)	173 (53.9)	35 (35.7)	

M—mean, SD—standard deviation.

**Table 2:** Nutritional status of type 1 diabetics, taking account of the place of residence.

Variables	Total (n = 419)	Urban Area (n = 321)	Rural Area (n = 98)	*p*-Value
BMI category—n (%)				
Underweight	5 (1.2)	3 (0.9)	2 (2.0)	0.014
Healthy Weight	216 (51.6)	179 (55.8)	37 (37.8)	
Overweight	192 (45.8)	134 (41.7)	58 (59.2)	
Obesity	6 (1.4)	5 (1.6)	1 (1.0)	

**Table 3:** Selected diabetes-related parameters of type 1 diabetics taking account of the place of residence.

Variables	Total (n = 419)	Urban Area (n = 321)	Rural Area (n = 98)	*p*-Value
HbA1c [%] M (SD)	7.5 (1.7)	7.4 (1.7)	7.8 (1.7)	0.013
Duration of diabetes—M (SD) [years]	18.8 (9.1)	19.2 (9.6)	17.5 (7.1)	0.396
Type of insulin therapy—n (%)				
Pens	226 (53.9)	173 (53.9)	53 (54.1)	0.974
Insulin pumps	193 (46.1)	148 (46.1)	45 (45.9)	
Insulin units per kilogram of body weight per day—n (%)				
<0.4	112 (26.7)	87 (27.1)	25 (25.5)	0.836
0.4–0.75	207 (49.4)	156 (48.6)	51 (52.0)	
>0.75	100 (23.9)	78 (24.3)	22 (22.4)	
Hypoglycemic episodes—n (%)				
Never	70 (16.7)	53 (16.5)	17 (17.3)	0.239
1–3 times a week	206 (49.2)	166 (51.7)	40 (40.8)	
4–6 times a week	121 (28.9)	87 (27.1)	34 (34.7)	
Every day	22 (5.3)	15 (4.7)	7 (7.1)	
Diabetes control based on hypoglycemic episodes—n (%)				
HbA1c ≤ 7%	276 (65.9)	219 (68.2)	57 (58.2)	0.066
HbA1c > 7%	143 (34.1)	102 (31.8)	41 (41.8)	
Hyperglycemic episodes—n (%)				
Never	45 (10.7)	34 (10.6)	11 (11.2)	0.921
1–3 times a week	127 (30.3)	100 (31.2)	27 (27.6)	
4–6 times a week	171 (40.8)	130 (40.5)	41 (41.8)	
Every day	76 (18.1)	57 (17.8)	19 (19.4)	
Number of glucose measurements daily—n (%)				
<5	103 (24.6)	70 (21.8)	33 (33.7)	0.001
5–9	221 (52.7)	185 (57.6)	36 (36.7)	
10 and more	95 (22.7)	66 (20.6)	29 (29.6)	

HbA1c—glycated hemoglobin, M—mean, SD—standard deviation.

**Table 4:** The use of psychological and psychiatric assistance/care by type 1 diabetics taking account of the place of residence.

Variables	Total (n = 419)	Urban Area (n = 321)	Rural Area (n = 98)	*p*-Value
Using psychological consultations—n (%)				
Yes	94 (22.4)	76 (23.7)	18 (18.4)	0.270
No	325 (77.6)	245 (76.3)	80 (81.6)	
Using psychiatric consultations—n (%)				
Yes	66 (15.8)	51 (15.9)	15 (15.3)	0.890
No	353 (84.2)	270 (84.1)	83 (84.7)	

**Table 5:** Psychological indicators and rate of adherence to dietary recommendations in type 1 diabetics taking account of the place of residence.

Variables	Total (n = 419)	Urban Area (n = 321)	Rural Area (n = 98)	*p*-Value
AIS—M (SD)	27.2 (8.2)	26.6 (8.1)	29.1 (8.2)	0.006
DDGA Index—M (SD)	17.7 (4.2)	18.0 (4.2)	16.5 (3.9)	0.001
DEPS-R Scale—M (SD)	16.7 (10.7)	16.1 (9.8)	18.7 (12.9)	0.206
EAT—M (SD)	10.1 (8.0)	10.0 (7.6)	10.4 (9.1)	0.810
SRHS—M (SD)	55.7 (7.7)	56.3 (7.5)	53.9 (8.1)	0.018

AIS—Acceptance of Illness Scale; DDGA Index—Diabetes Dietary Guidelines Adherence Index; DEPS-R—Diabetes Eating Problem Survey-Revised Scale; EAT—Eating Attitudes Test; SRHS—Sense of Responsibility for Health Scale; M—mean; SD—standard deviation.

## Data Availability

The data presented in our study are available upon request from the corresponding author.
